# Decoupling growth phase dependency and metal ion inhibition: A dual engineering strategy for the high-yield biosynthesis of microcin J25 in *Escherichia coli*

**DOI:** 10.1016/j.engmic.2025.100230

**Published:** 2025-08-14

**Authors:** Guangxin Yang, Xinchan Wang, Yunting Zhou, Xiuliang Ding, Jinxiu Huang, Shiyan Qiao, Aihua Deng, Haitao Yu

**Affiliations:** a State Key Laboratory of Animal Nutrition and Feeding, Ministry of Agriculture and Rural Affairs Feed Industry Centre, China Agricultural University, Beijing 100193, China.; b Frontier Technology Research Institute of China Agricultural University in Shenzhen, Shenzhen 518119, China; c Chongqing Academy of Animal Science, Rongchang, Chongqing 40240, China; d National Center of Technology Innovation for Pigs, Rongchang, Chongqing 40240, China.

**Keywords:** Antimicrobial peptide, Microcin J25, Recombinant expression, Biosynthesis

## Abstract

Microcin J25 (MccJ25) has received substantial attention as a potential solution to the global threat of infection caused by antibiotic-resistant bacteria. However, the industrial fermentation of MccJ25 faces production bottlenecks. It is imperative to further explore the production optimization strategies for MccJ25 to formulate comprehensive approaches for its industrial-scale production and other downstream applications. Here, Fe²⁺ in tap water was identified as a critical inhibitor of MccJ25 biosynthesis, selectively repressing *mcjA* transcription, which was reversible via 2,2′-bipyridine-mediated chelation. To decouple production from growth phase dependency and Fe²⁺ interference, we engineered *Escherichia coli* BL21 cells by performing two genetic modifications. First, we replaced the native *mcjA* promoter with a constitutive promoter (P_Q_) to allow its mid-log phase expression. Second, we replaced the native *mcjBCD* promoter with a medium-strength variant (P_2223_) that delayed production kinetics without affecting final yields. However, the genomic integration of *mcjD* alleviated plasmid-borne toxicity, increasing the expression timing and doubling the yield to 240 mg/L. Finally, we computationally optimized the *mcjA* ribosome-binding site (RBS) to enhance translation efficiency. RBS optimization revealed that a moderate translation initiation efficiency (550,584 arbitrary units [au]) maximized production, whereas excessive efficiency (2,019,712 au) impaired growth and output. These interventions synergistically increased the MccJ25 titer 10-fold, reaching 430 mg/L in batch culture. Our findings establish a robust platform for MccJ25 overproduction, highlighting promoter engineering and translational tuning as pivotal strategies for antimicrobial peptide biosynthesis. This study provides insights for overcoming metabolic constraints in microbial fermentation, advancing the development of peptide-based therapeutics against multidrug-resistant pathogens.

## Graphical Abstract

**Figure:**
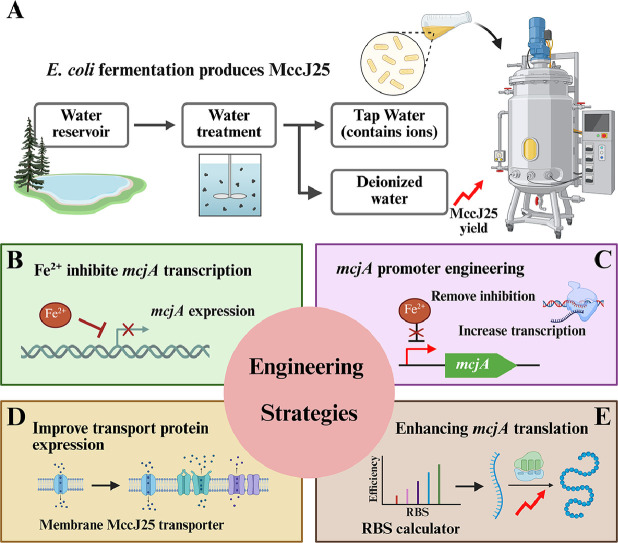

## 1 Introduction

The emergence of antibiotic resistance is one of the most challenging global health crises because the overadministration of antibiotics has resulted in an increase in therapeutic failure rates for various infectious diseases [1]. The development of new drugs in the 21^st^ century has slowed considerably, while advanced antibiotic development approaches such as genomics, high-tech chemical synthesis, and high-throughput screening have been unsuccessful in discovering new antibiotics, particularly those used for the treatment of gram-negative enteric bacteria [2,3]. Therefore, there is an increasing need for new strategies to combat antibiotic resistance. In recent years, a range of alternative therapies to traditional antibiotics has been developed, including probiotics, antiviral molecules, antibodies, nano/micromaterials, and antimicrobial peptides (AMPs) [4]. AMP-based biomaterials have gained considerable attention owing to their ability to overcome multidrug resistance and provide excellent templates for a wide range of bioengineering and biomedical applications, including regenerative medicine, biomimetic materials, antimicrobial agents, and therapeutic drug delivery [5,6].

Microcins are a class of ribosomally synthesized AMPs encoded by genetic systems with a conserved structural organization [7]. These peptides have garnered increasing research attention because of their unique biochemical and structural properties, mechanisms of action, potent antimicrobial activity, and immunomodulatory effects [8,9]. Microcin J25 (MccJ25), a low-molecular-weight, plasmid-encoded, ribosomally synthesized AMP, was initially isolated from a fecal-derived *Escherichia coli* strain [10]. Its exceptional stability, conferred by its unique lasso-shaped tertiary structure [11], has positioned MccJ25 as a promising scaffold for peptide-based bioengineering applications. The genetic characterization of MccJ25 biosynthesis revealed that its operon comprises four genes (*mcjA, mcjB, mcjC*, and *mcjD*), each under distinct transcriptional control: *mcjA* is driven by an independent promoter, whereas *mcjB, mcjC*, and *mcjD* share a bidirectional promoter upstream of *mcjB* [12]. Temporal expression profiling demonstrated that *mcjB* and *mcjC* are transcribed during the exponential growth phase, whereas *mcjA* is exclusively activated during the stationary phase under nutrient-limiting conditions (carbon and inorganic phosphate starvation), enabling high-titer MccJ25 production [13]. Notably, substituting the native *mcjA* promoter with the inducible P_T5_ promoter facilitated early-phase expression; however, despite elevated *mcjA* mRNA levels following IPTG induction, MccJ25 yields remained suboptimal [14]. In a previous study, we established a heterologous expression system for MccJ25 using the engineered plasmid pMJ25 by leveraging advanced recombinant DNA methodologies [15,16]. Nevertheless, a systematic investigation into the effects of synthetic operon design on the efficiency of MccJ25 biosynthesis remains imperative.

In the current study, we identified Fe²⁺-mediated transcriptional repression of *mcjA* as a critical bottleneck limiting MccJ25 titers in industrial fermentation processes. To decouple production from growth phase dependency and Fe²⁺ interference, we implemented a dual engineering strategy through: (i) replacement of the native *mcjA* promoter with a constitutive promoter Q (P_Q_) to ensure continuous transcription and (ii) computational redesign of the *mcjA* ribosome-binding site (RBS) to maximize translational efficiency. These synergistic modifications resulted in a 10-fold increase in MccJ25 yield, resulting in a final concentration of 430 mg/L in the batch culture.

## 2 Materials and methods

### 2.1 Bacterial strains and growth conditions

The bacterial strains and plasmids used in this study are listed in Table S1. Luria–Bertani (LB) medium (10 g/L NaCl, 10 g/L tryptone, and 5 g/L yeast extract) was used for cloning *E. coli* DH5α and the fermentation of MccJ25. Appropriate antibiotics, such as 100 μg/mL ampicillin (Amp), 50 μg/mL kanamycin (Kana), 25 μg/mL tetracycline (Tet), and 10 μg/mL chloramphenicol, were used for the selection of transformants when necessary. All the strains were cultured at 37 °C and 220 rpm unless otherwise specified.

### 2.2 Engineered gene cluster design for the biosynthesis of MccJ25

The primers and amplified fragments used for the construction of the recombinant plasmids used in the present study are listed in Table S2. The MccJ25 gene cluster (**AF061787**) [17] was synthesized by Sangon Biotechnology (Shanghai, China).

**Fig. 1:**
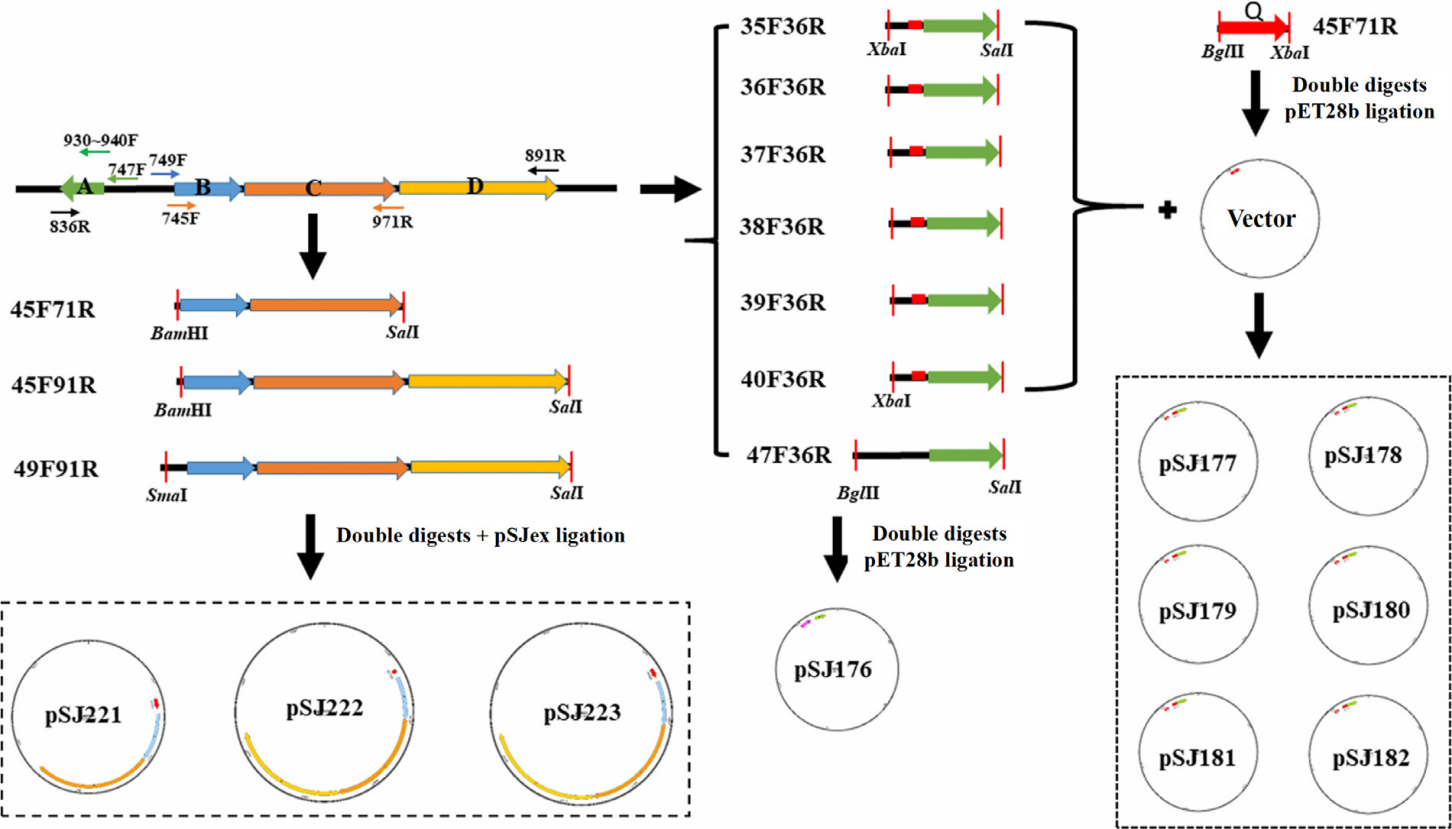
Construction of recombinant expression vectors for *mcjA* and *mcjBCD*. The wild-type *mcjABCD* expression cassette was inserted into the vector pET28b to construct plasmid pSJ176. Promoter Q and *mcjA*, which have different ribosome-binding site sequences, were inserted into vector pET28b to construct plasmids pSJ177–pSJ182. The *mcjBC* and *mcjBCD* expression cassettes were inserted into the vector pSJex to construct plasmids pSJ221, pSJ222, and pSJ223.

To generate the *mcjA* expression system, the Q promoter (P_Q_) was inserted into pET28b via *Bgl*II/*Xba*I digestion. Five *mcjA* variants (35F–40F/36R), which were amplified from the wild-type MccJ25 genome using primers 935F–940F/836R, respectively, were subsequently cloned downstream of the Q promoter through *Xba*I/*Sal*I ligation, generating the plasmids pSJ178–pSJ182. These included wild-type RBS (pSJ177) and five synthetic RBS variants (pSJ178–pSJ182). The native *mcjA* gene cassette (pSJ176) was generated by inserting a *Bgl*II/*Sal*I-digested 443-bp fragment (47F36R, amplified using primers 747F/836R) into pET28b. For *mcjBCD* expression, a 4,042-bp fragment (49F91R, primers 749F/891R) was cloned and inserted into pSJex via *Sma*I/*Sal*I restriction to create pSJ223 harboring the native *mcjB* promoter, whereas a 3,973-bp fragment (45F91R, primers 945F/891R) was inserted via *BamH*I/*Sal*I to generate pSJ222 with the medium-strength promoter P_2223_. The *mcjBC* expression vector pSJ221 was constructed by ligating a *BamH*I/*Sal*I-digested 2,214-bp fragment (45F71R, primers 945F/971R) into pSJex, positioning *mcjBC* downstream of the P_2223_ promoter. All cloning procedures were performed in *E. coli* DH5α cells cultured in LB broth for plasmid amplification. All constructs were sequence-verified, and the complete cloning strategy is illustrated in Fig. 1.

### 2.3 Fermentation and quantitative evaluation of MccJ25 expression

Purified *E. coli* colonies expressing MccJ25 were precultured in LB medium and incubated at 37 °C with shaking at 220 rpm for 16 h to obtain the seed cultures. 1 mL of the seed culture mixture was inoculated into 50 mL of LB medium in a 250 mL flask and cultured at 37 °C with shaking at 220 rpm for 24 h. Kanamycin (50 μg/mL) and ampicillin (100 μg/mL) were supplemented as needed.

For MccJ25 analysis, 2 mL of fermentation broth was centrifuged at 8,000 × *g* for 5 min at 4°C to collect the supernatant, which was then filtered through a 0.22-μm membrane. MccJ25 was qualitatively and quantitatively analyzed using an Agilent 1260 reversed-phase high-performance liquid chromatography (RP-HPLC) system (Agilent, USA). The detailed detection methods and parameters are listed in Table S3.

Pure MccJ25 sample was prepared as described in prior studies [16].

### 2.4 mRNA extraction and transcript analysis

The mRNA levels of *mcjA, mcjB, mcjC*, and *mcjD* in the MccJ25-expressing strains were analyzed using RT-PCR. The primers used for real-time PCR are listed in Table 1. Briefly, total RNA from *E. coli* cells was extracted and purified using the TRIzol reagent (Invitrogen, USA) according to the manufacturer’s protocol. The purity and yield of the extracted RNA were evaluated via a NanoDrop2000 (Thermo, USA). Serial 10-fold dilutions of plasmid standards (pSJ172, pSJ173, pSJ174, and pSJ175, 10^0^−10^−7^) were prepared, with triplicates for each dilution. A standard curve was generated by plotting the log_10_ plasmid copy numbers against the Ct values. The amplification efficiency was calculated as E=10^(−1/Slope)^ −1, copy numbers were calculated from standard curves (R^2^ > 0.98), and outliers were excluded if the technical replicates presented > ± 0.5 Ct variation.

**Table 1:** RT-PCR primers.

Gene name	Sequences (5′–3′)	Size (bp)
*mcjA*	F-AGCCCGGCAAAAGGTGTTATTCAG	179
	R-ACCCGGACCCACGAAATACTCC	
*mcjB*	F-ATCTGACTGTCTTACCTATTCATACGC	219
	R-CCCAACCTCCACCCAAGAGT	
*mcjC*	F-CAACCCAGGAATCACTAA	198
	R-TCCCGCATCATCTCAATA	
*mcjD*	F-GGACTGGTTTTCTGCCGGTGTG	204
	R-TCCATGCTGTGTTTTCTGAGAGACG	

Note: F, forward primer; R, reverse primer.

### 2.5 Construction of genome-integrated expression strains

The primers and amplified fragments used for the construction of the recombinant plasmids used in the present study are listed in Table S2.

The BL21Cas strain (harboring the pCas plasmid for RED recombinase induction and constitutive Cas9 expression) was used for genomic integration. Competent cells were prepared in Luria–Bertani (LB) medium supplemented with 10 mM arabinose. The linear DNA fragment 1102FR was amplified from pTN52 using the primers 1102F/1102R. Co-transformation with the pThsds plasmid was performed via heat shock (42°C, 90 s), followed by recovery in LB and plating on Amp/Tet plates. Positive clones were verified using PCR (primers 1103F/1103R).

Plasmid curing was also conducted. Positive clones were cultured in Tet-containing LB medium, serially passaged, and plated onto Tet plates. Colonies that did not grow on the Amp/Kana plates were designated as BL21(1102FR).

To eliminate tetracycline resistance, BL21(1102FR) cells were transfected with pDZCrc. Colonies relieved of Tet resistance (those that did not grow on the Tet plates) were named BL21D.

Regarding expression strain construction, plasmids pSJ177, pSJ182, and pSJ221 were co-transformed into BL21D, generating the expression strains BL21Ex1–Ex6 (Table S1).

### 2.6 Statistical analyses

Two-tailed Student’s t-tests were used for single comparisons, whereas analysis of variance (ANOVA) with Tukey’s test was used for multiple comparisons. *P* < 0.05 was considered statistically significant. Statistical analyses and graphing were performed using GraphPad Prism 9 software. The data are presented as the means ± standard errors of the means unless otherwise stated.

## 3 Results

### 3.1 Fe^2+^ reduces the production of MccJ25 by inhibiting *mcjA* transcription

**Fig. 2:**
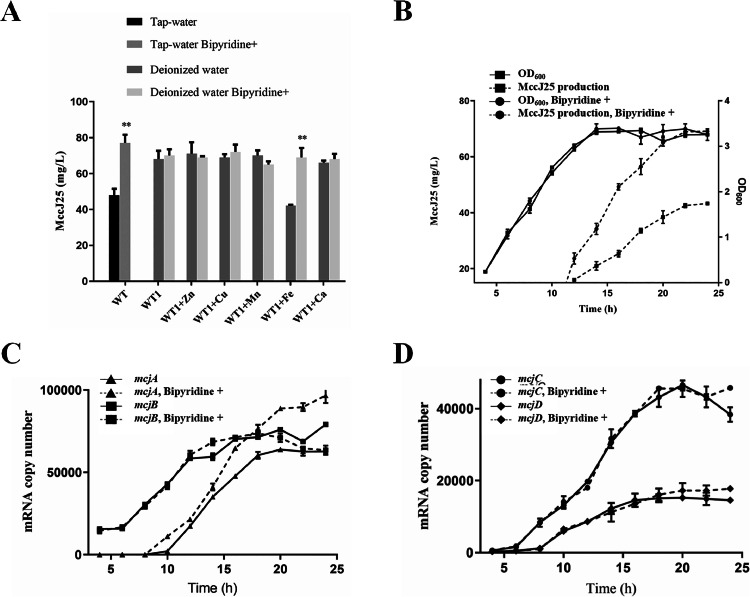
Fe^2+^ in tap water inhibits the expression of *mcjA.* (A) Effect of Luria–Bertani (LB) medium prepared with tap water versus LB medium prepared with deionized water on MccJ25 production by the wild-type (WT) strain. Zn²⁺, Cu²⁺, Mn²⁺, Fe²⁺, and Ca²⁺ indicate the addition of 10 mM of the corresponding metal ions to the media. (B) Growth curve and MccJ25 expression profile of the WT strain cultured in LB medium prepared with tap water. (C, D) mRNA copy numbers of *mcjA, mcjB, mcjC*, and *mcjD* in the MccJ25 WT strain. “Bipyridine^+^” denotes the addition of 20 μg/mL 2,2′-bipyridine to the medium. All experiments were independently repeated three times. Data are presented as the mean ± standard error of the mean. ** indicates highly significant differences compared with the control group (*P* < 0.01).

The effect of tap water on MccJ25 biosynthesis in wild-type (WT) *E. coli* was investigated by comparing MccJ25 yields in LB media prepared with tap water to those in LB media prepared with deionized water. As shown in Fig. 2A, MccJ25 production was significantly higher in LB medium prepared with deionized water than that prepared with tap water (*P* < 0.05). To address whether metal ions in tap water mediate this suppression, 2,2′-bipyridine—a bidentate chelator with high affinity for divalent metal ions—was introduced into the media. Strikingly, supplementation with 2,2′-bipyridine rescued MccJ25 production in tap water-based media to levels comparable to those of the deionized water controls (*P* < 0.05), whereas no effect was observed in the deionized water-based media. These results suggested that tap water contains metal ions that specifically inhibit MccJ25 biosynthesis in the WT strain.

To pinpoint the inhibitory metal species, Zn²⁺, Cu²⁺, Mn²⁺, Fe²⁺, and Ca²⁺ were individually added to deionized water in LB media (final concentration: 10 mM). Among these, only Fe²⁺ significantly suppressed MccJ25 production, reducing yields by approximately 40% (*P* < 0.05, Fig. 2A). Notably, the coaddition of 2,2′-bipyridine with Fe²⁺ fully restored MccJ25 expression, confirming that Fe²⁺ is the primary inhibitory metal ion in tap water. In contrast, Zn²⁺, Cu²⁺, Mn²⁺, and Ca²⁺ had no discernible effect on MccJ25 production levels.

Further experiments characterized the dual effects of Fe²⁺ on bacterial growth and MccJ25 expression. The growth kinetics and temporal expression profiles of the WT strain were analyzed in tap water- or deionized water-prepared LB media with or without 2,2′-bipyridine. As shown in Fig. 2B, tap water had no detectable effect on the growth dynamics or the timing of MccJ25 expression initiation (occurring during the stationary phase). However, MccJ25 titers in the tap water media lacking bipyridine remained consistently lower than those in the bipyridine-supplemented media throughout the cultivation period (*P* < 0.05). These findings collectively demonstrate that Fe²⁺ in tap water selectively inhibits MccJ25 biosynthesis without perturbing cellular growth. This inhibition was reversible through metal chelation.

Bacterial cells were collected every 2 h from the early logarithmic to the stationary phase based on the growth curve and the MccJ25 expression profile of the WT strains. The expression levels of the MccJ25 biosynthesis genes *mcjA, mcjB, mcjC*, and *mcjD* were quantified via qPCR. As shown in Figs. 2C and 2D, *mcjA* transcription was initiated prior to entry into the stationary phase, whereas *mcjB, mcjC*, and *mcjD* were expressed during the logarithmic phase, with *mcjB* exhibiting significantly higher transcript levels than *mcjC* and *mcjD* (*P* < 0.05). Notably, supplementation with 2,2′-bipyridine in tap water-prepared LB medium markedly increased *mcjA* expression without affecting *mcjB, mcjC*, or *mcjD* (Fig. 2C and 2D). These findings indicate that MccJ25 production is synchronized with *mcjA* transcription, with both initiating upon entry into the stationary phase, whereas Fe²⁺ suppresses MccJ25 biosynthesis by inhibiting *mcjA* transcription.

### 3.2 Reengineered and reconstructed gene clusters produce biosynthetic MccJ25

To verify the relationship between the natural promoters of *mcjA* and F^2+^, we constructed a series of plasmids. The *mcjA* gene is controlled by a constitutive promoter (P_Q_) when used to replace the growth state-dependent promoter. However, expression of the naturally controlled *mcjBCD* gene cluster remained unchanged.

**Fig. 3:**
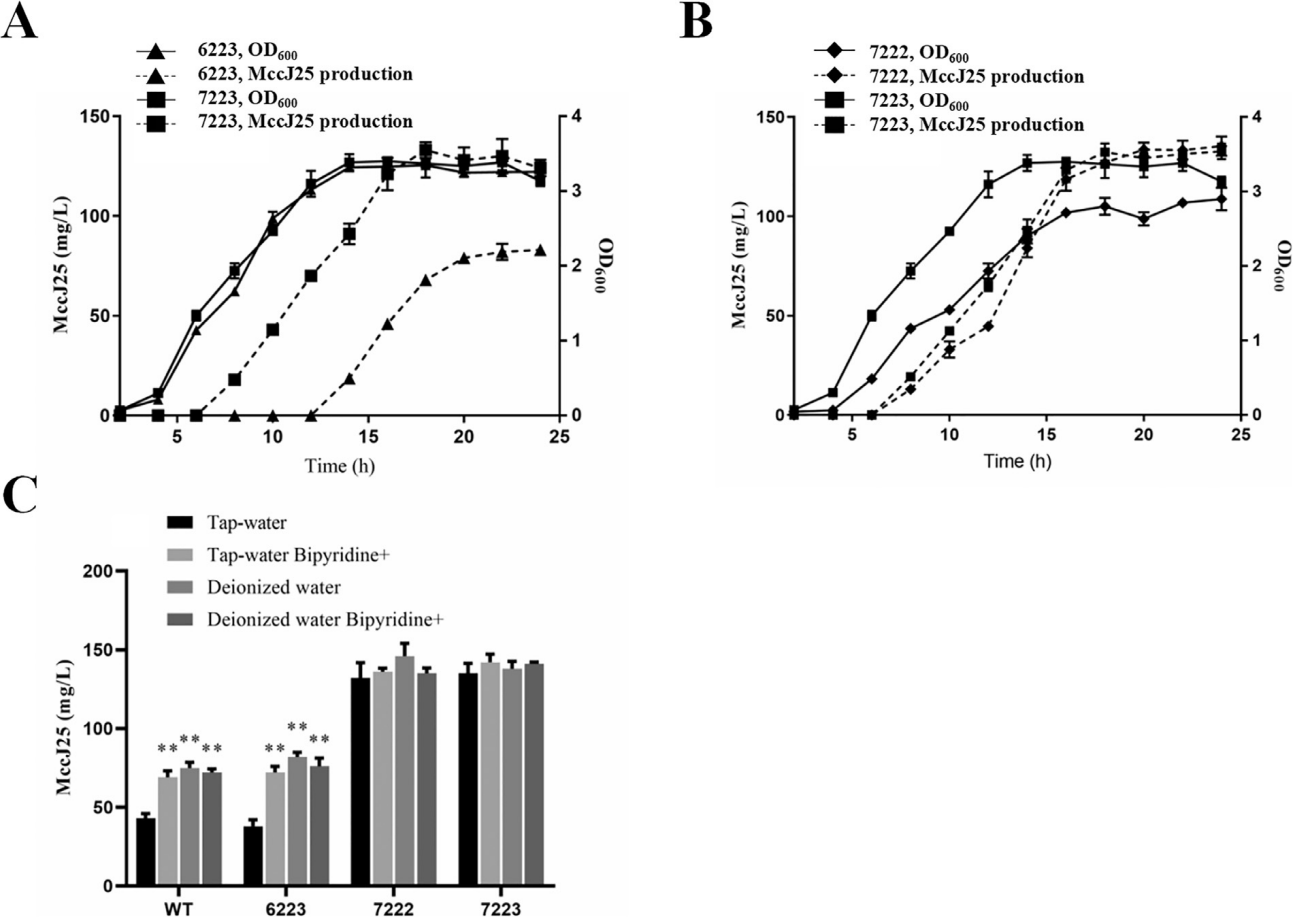
*mcjABCD* promoter replacement promotes the synthesis of MccJ25. Effect of replacing the (A) *mcjA* promoter with P_Q_ and (B) *mcjBCD* promoter with P2223 on the growth performance of the strain and the yield of MccJ25. (C) Effects of tap water- and deionized water-prepared culture media on MccJ25-expressing strains. Strain numbers: 6222, *E. coli* BL21 containing natural *mcjA* and *mcjBCD* gene clusters; 7222, *E. coli* BL21 containing P_Q_-*mcjA* and P_2223_-*mcjBCD*; and 72223, *E. coli* BL21 containing P_Q_-*mcjA* and WT-*mcjBCD*. “Bipyridine^+^” denotes the addition of 20 μg/mL 2,2′-bipyridine to the medium. All experiments were independently repeated three times. Data are presented as the mean ± standard error of the mean. ** indicates highly significant differences compared with the control group (*P* < 0.01).

The production of MccJ25 was detected in the fermentation broth of the recombinant expression strain of *E. coli* BL21 (Fig. S1), and its molecular composition was confirmed using liquid chromatography-mass spectrometry (LC-MS) (Fig. S2). As shown in Fig. 3A, MccJ25 was expressed by 7223 (pSJ177, containing engineered *mcjA* and natural *mcjBCD* genes) in the mid-log phase of growth and by 6223 (pSJ176, containing natural *mcjA* and *mcjBCD* gene clusters) in the stationary phase of growth. Furthermore, the titer of MccJ25 increased during the transition from mid-log to stationary phase.. These findings indicated that the expression of MccJ25 was independent of the growth state and that the inhibitory effect of iron ions on the expression of MccJ25 was abrogated under the control of the P_Q_ promoter.

To determine whether the promoter that controls the expression of the *mcjBCD* gene could also affect the expression of MccJ25, we constructed a pSJ222 plasmid controlled by the medium-strength promoter P_2223_ to express the *mcjBCD* gene cluster. Thereafter, pSJ177 (control, engineered *mcjA* expression) and pSJ222 were transformed into *E. coli* BL21 cells and designated as 7222. The expression titers of MccJ25 and the OD_600_ of the recombinant bacteria were determined together with 7223. As shown in Fig. 3B, the production of MccJ25 lagged under the control of the promoter P_2223_. Additionally, the growth rate of the recombinant bacteria decreased, as evidenced by the low OD_600_ value in the stationary phase of growth, when the *mcjBCD* gene was under the control of the medium-strength promoter. However, the expression titer of MccJ25 under the control of the P_2223_ promoter was similar to that of MccJ25 under the control of a wild-type promoter.

A comparative analysis was then conducted to evaluate the effects of tap water and LB medium on MccJ25 production in strains MccJ25 WT, 6222, 7222, and 7223. As shown in Fig. 3C, strain 6222 (harboring wild-type *mcjABCD*) exhibited MccJ25 expression levels comparable to those of the WT MccJ25 strain, with expression suppressed by metal ions present in tap water. This suppression was reversed by bipyridine supplementation, which restored MccJ25 production in the tap water-based media. Notably, strains 7223 and 7222 (both carrying substituted *mcjA* promoters) showed elevated MccJ25 expression and were unaffected by metal ions in tap water. Furthermore, the dual substitution of the *mcjA* and *mcjBCD* promoters in strain 7222 did not confer additive effects on MccJ25 expression compared to strain 7223 (which contained only a modified *mcjA* promoter).

### 3.3 The recombinant *mcjD* expression cassette enhances the biosynthesis of MccJ25

**Fig. 4:**
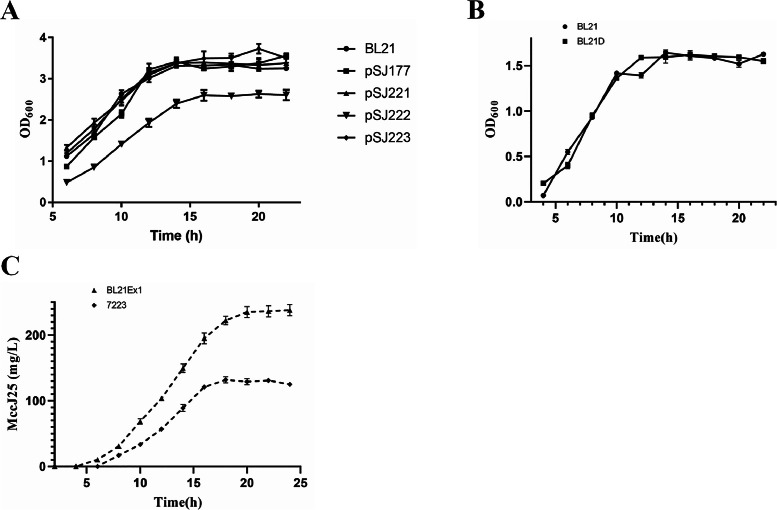
The recombinant expression of *mcjD* promoted the biosynthesis of MccJ25. (A) Growth curve of the MccJ25 recombinant expression strain. (B) Growth curve and MccJ25 production of *E. coli* BL21 after the integration of the P_2223_-*mcjD* expression cassette into the genome. BL21D, *E. coli* BL21 contains P_Q_-*mcjA* and P_2223_-*mcjBC* on the plasmid, and P_2223_-*mcjD* on the genome; BL21Ex1, *E. coli* BL21 containing P_Q_-*mcjA* and P_2223_-mcjBC on the plasmid, and P_2223_-*mcjD* on the genome; and 72223, *E. coli* BL21 containing P_Q_-*mcjA* and WT-*mcjBCD*. All experiments were independently repeated three times. Data are presented as the mean ± standard error of the mean.

A previous study reported that the overexpression of membrane proteins can be toxic to the host [18]. In the natural MccJ25 gene cluster, *mcjD* belongs to a family of membrane proteins [19]. Therefore, we hypothesized that high *mcjD* expression may decrease the growth rate of recombinant bacteria. To test this hypothesis, we constructed the pSJ221 plasmid to induce the expression of the *mcjBC* gene under the control of the promoter P_2223_. Thereafter, a series of plasmids (pSJ177, pSJ221, pSJ222, and pSJ223) were transformed into *E. coli* BL21 cells, and a growth curve was established for the recombinant bacteria. As shown in Fig. 4A, the growth of pSJ177, pSJ221, and pSJ223 was similar to that of untransformed *E. coli* BL21; however, the growth of the pSJ222 strain was significantly delayed. These results indicate that the large-scale expression of *mcjD* severely affects the growth of recombinant bacteria.

The expression of *mcjD* in plasmids may lead to overexpression because plasmids may have a high copy number. Therefore, *mcjD* was integrated into the *hsdS* locus of the *E. coli* BL21 genome (strain BL21D) to reduce the copy number of *mcjD*. The results are shown in Fig. 4B. The growth curve of strain BL21D, with the P_2223_-*mcjD* expression cassette integrated into its genome, was similar to that of *E. coli* BL21. Notably, compared to strain 7223, the expression duration of MccJ25 in strain BL21Ex1 was greater, and the yield increased two-fold to 240 mg/L (Fig. 4C).

### 3.4 Engineering the RBS to enhance mcjA translational efficiency

The synthesis rate of translation initiation regulatory proteins [20] suggests that enhancing MccJ25 production requires not only elevated mRNA levels but also improved translational efficiency. In this study, five RBSs for the *mcjA* gene were computationally designed using an RBS calculator, with predicted translation initiation efficiencies of 90,504, 243,009, 550,584, 1,231,097, and 2,019,712 au. The corresponding plasmid constructs (pSJ178, pSJ179, pSJ180, pSJ181, and pSJ182, harboring RBS1-RBS5, respectively) were generated. The plasmids pSJ177 and pSJ178-pSJ182 were co-transformed with pSJ221 into strain BL21D, yielding expression strains BL21Ex2–BL21Ex6. Notably, BL21Ex1 retained the wild-type *mcjA* RBS with a translation initiation efficiency of 23.587 arbitrary units (au), which was significantly lower than that of RBS1–RBS5.

**Fig. 5:**
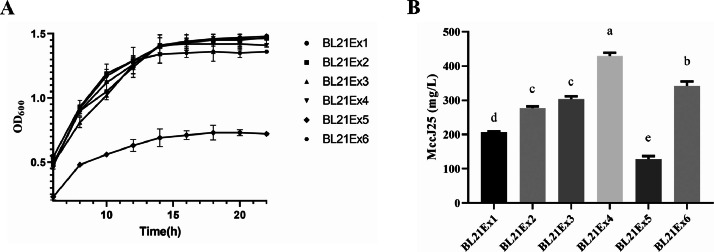
Effect of initial *mcjA* translation efficiency on the expression of MccJ25. (A) Growth curves and (B) MccJ25 production of *mcjA*-expressing strains with different initial translation efficiencies. All experiments were independently repeated three times. Data are presented as the mean ± standard error of the mean. Different lowercase letters indicate significant differences between means (*P* < 0.05).

As shown in Fig. 5, compared with the other strains, BL21Ex5 had the highest translation initiation efficiency, but its MccJ25 expression was the lowest. Moreover, the growth rate of BL21Ex5 was lower than that of the other strains. The growth rate of BL21Ex6 and the expression level of MccJ25 decreased after the cells entered the stationary phase of growth. Surprisingly, the growth rate of BL21Ex4 was similar to that of BL21Ex1; however, the MccJ25 titer in BL21Ex4 reached 430 mg/L, which was greater than that in BL21Ex1. To evaluate the industrial production capacity of the recombinant *mcjABCD* gene cluster, scaled-up fermentation of strain BL21Ex4 was conducted in a 50 L bioreactor. Under controlled conditions, fed-batch fermentation enabled sustained MccJ25 expression, significantly enhancing the yield to 2,440 mg/L (Fig. S3).

## 4 Discussion

The biosynthesis of MccJ25, a lasso-shaped antimicrobial peptide with therapeutic potential, is constrained by growth phase dependency, environmental metal ion interference, and host metabolic burden. Our study identified Fe²⁺ in tap water as a critical inhibitor of *mcjA* transcription, reducing MccJ25 yields by 40% (*P* < 0.05), a repression reversible through 2,2′-bipyridine chelation. While previous studies have linked MccJ25 production to nutrient starvation, our findings highlight Fe²⁺ as an industrial-scale bottleneck, emphasizing the need for water quality control in fermentation processes. To decouple production from growth phase limitations, we engineered *Escherichia coli* BL21 by replacing the native *mcjA* promoter with a constitutive promoter (P_Q_), enabling mid-log phase expression and overriding Fe²⁺-mediated repression. This modification synchronized *mcjA* transcription with the expression of *mcjBCD*-encoded maturation enzymes, streamlined precursor availability, and resulted in a 10-fold increase in yield (430 mg/L).

The biosynthesis, maturation, and secretion of MccJ25 require the coordinated involvement of *mcjABCD*, with *mcjABC* responsible for synthesis and processing, and *mcjD* for mediating transport [21]. The synergistic interactions of these genes govern the dynamics of MccJ25 expression. Previous studies demonstrated that MccJ25 production is strictly regulated by the bacterial growth phase because of the stationary phase-dependent transcription of *mcjA*, which is exclusively induced after nutrient depletion [13]. Our findings corroborate this observation, showing a significant elevation in *mcjA* mRNA levels, coinciding with the MccJ25 expression profiles during the stationary phase (Fig. 2). Notably, in addition to growth phase regulation, *mcjA* transcriptional activity was substantially suppressed by Fe²⁺, resulting in an approximately 40% reduction in MccJ25 protein yield (Fig. 2). Previous studies demonstrated that using the *lac* operator to control the T5 promoter to induce *mcjA* expression eliminates the growth phase and nutrient limitation dependencies of the native MccJ25 gene cluster in *E. coli* BL21. This approach increased MccJ25 production to 8 mg/L in LB medium (1.5- to 2-fold enhancement) [14]. However, whether this modification affects Fe²⁺-mediated transcriptional repression remains unclear. In this study, we engineered the *mcjA* promoter to decouple MccJ25 production from both growth phase dependency and Fe²⁺ suppression. Consequently, the yield of MccJ25 reached 130 mg/L in the LB medium prepared with tap water, representing a significant increase from the baseline yield of 40 mg/L. This inhibition has critical industrial implications because the pipeline and fermenter systems contain abundant iron ions. Even media prepared with deionized water failed to eliminate iron-mediated suppression while incurring prohibitive costs, underscoring the necessity of mitigating Fe²⁺-mediated gene expression inhibition. Our data revealed that Fe²⁺ suppresses MccJ25 biosynthesis by inhibiting *mcjA* transcription (Fig. 2C), an effect completely abrogated through the promoter replacement of *mcjA*.

The *mcjBCD* operon facilitates MccJ25 maturation. While prior studies observed altered MccJ25 expression upon the inducible promoter-driven regulation of *mcjBCD*, yields remained unchanged (≤ 8 mg/L) compared to the WT expression cassette [14]. Similarly, our data revealed that *mcjBCD* expression driven by moderate-strength promoters failed to enhance MccJ25 production and impaired bacterial growth. Given the typical positive correlation between *E. coli* growth and metabolite yield, we hypothesized that increased *mcjBCD* transcriptional activity stimulates MccJ25 biosynthesis. Notably, *mcjD* encodes a membrane-localized transporter essential for MccJ25 secretion [19]. Its overexpression induces membrane toxicity via an occupancy effect that compromises membrane fluidity [22]. Our experiments demonstrate that plasmid-mediated *mcjD* overexpression severely inhibits host growth. In contrast, chromosomal integration eliminated this growth defect, while boosting the MccJ25 yield to 230 mg/L (Fig. 4). This outcome underscores the toxicity of mcjD membrane transporter overexpression and the utility of genomic integration for controlled expression.

The RBS serves as a critical regulatory element that directs efficient and precise ribosome-mRNA engagement during translation initiation [20]. Variations in the secondary structures formed between the coding sequences and RBSs modulate translational efficiency [23], making RBS engineering an effective approach for enhancing metabolite production through optimized protein expression [24]. The RBS Calculator software employs a biophysical model based on thermodynamic parameters and free-energy calculations that can accurately predict the translation start rate of the RBS [25]. We achieved 430 mg/L of MccJ25 production through medium-strength RBS optimization for *mcjA* expression. However, further increases in RBS translational efficiency compromised cellular growth (Fig. 5), likely because of ribosomal resource competition. Highly efficient RBS sequences coupled with strong promoters may sequester limited ribosomes, thereby impairing global protein synthesis [26].

## 5 Conclusion

In conclusion, this work elucidated the Fe²⁺-mediated transcriptional repression of *mcjA* as a critical bottleneck in MccJ25 biosynthesis. The strategic replacement of the native *mcjA* promoter with its constitutive counterpart effectively uncoupled production from Fe²⁺-mediated inhibition and growth phase dependency. Furthermore, the synergistic integration of RBS optimization for *mcjA* and genomic *mcjD* stabilization resulted in unprecedented MccJ25 titers (430 mg/L), establishing a new benchmark for ribosomal peptide synthesis. These breakthroughs not only unravel key regulatory constraints but also provide a paradigm for industrial biosynthesis optimization through multilevel engineering of transcriptional control, transport efficiency, and translational precision.

## Data Availability Statement

All relevant data supporting the findings of this study are available in this manuscript and the supplementary materials.

## CRediT authorship contribution statement

**Guangxin Yang:** Writing – original draft, Formal analysis, Data curation. **Xinchan Wang:** Writing – original draft, Formal analysis, Data curation. **Yunting Zhou:** Formal analysis, Data curation. **Xiuliang Ding:** Methodology. **Jinxiu Huang:** Methodology, Funding acquisition. **Shiyan Qiao:** Funding acquisition, Conceptualization. **Aihua Deng:** Writing – review & editing, Supervision, Methodology. **Haitao Yu:** Writing – review & editing, Supervision, Funding acquisition, Conceptualization.

## Declaration of Competing Interest

The authors declare the following financial interests/personal relationships which may be considered as potential competing interests: Haitao Yu reports administrative support was provided by China Agricultural University.

## Appendix Supplementary materials

Image, application 1
