# Supplementary material for: Decoupling growth phase dependency and metal ion inhibition: A dual engineering strategy for the high-yield biosynthesis of microcin J25 in Escherichia coli
Source: Eng Microbiol. 2025 Aug 14;5(4):100230. doi: 10.1016/j.engmic.2025.100230 (PMC12967822; doi:10.1016/j.engmic.2025.100230)
Supplement: Supplementary file 1 [file mmc1.docx]

**Supplementary Materials**

**Decoupling Growth-Phase Dependency and Metal Ion Inhibition: A Dual Engineering Strategy for High-Yield Biosynthesis of Microcin J25 in *Escherichia coli***

Guangxin Yang^1,2,a^, Xinchan Wang^1,2,a^, Yunting Zhou^1,2^, Xiuliang Ding^3,4^, Jinxiu Huang^3,4^, Shiyan Qiao^1,2^, Aihua Deng^1,2,^* and Haitao Yu^1,2,^*

^1^State Key Laboratory of Animal Nutrition and Feeding, Ministry of Agriculture and Rural Affairs Feed Industry Centre, China Agricultural University, Beijing 100193, PR. China.

^2^Frontier Technology Research Institute of China Agricultural University in Shenzhen, Shenzhen, 518119, China;

^3^Chongqing Academy of Animal Science, Rongchang, Chongqing 40240, China;

^4^National Center of Technology Innovation for Pigs, Rongchang, Chongqing 40240, China.

* Corresponding author: Aihua Deng (Deng A. H.), E-mail: [dengah@cau.edu.cn](mailto:dengah@cau.edu.cn); Haitao Yu (Yu H. T.), E-mail: [yuhaitao@cau.edu.cn](mailto:yuhaitao@cau.edu.cn).

^a^ These authors contributed equally to the study.

State Key Laboratory of Animal Nutrition and Feeding, China Agricultural University, No. 2 Yuanmingyuan West Road, Haidian District, Beijing 100193, PR China.

**Table S1. Strains and plasmids used in this study.**

| Items | Descriptions | Sources |
| --- | --- | --- |
| Strains |  |  |
| MccJ25 WT |  | Our Lab |
| *E. coli* DH5α | Used for plasmid construction | Sangon Biotech |
| BL21 | Used for MccJ25 recombinant expression | Sangon Biotech |
| BL21Cas | *E. coli* BL21 contain the plasmid pDZcas | This work |
| BL21(1102FR) | BL21 hsdSB(rB-mB-)::1102FR | This work |
| BL21(BCD) | BL21 contain pSJ183 | This work |
| BL21(BC) | BL21 contain pSJ184 | This work |
| BL21(B) | BL21 contain pSJ185 | This work |
| BL21D | BL21 hsdSB(rB-mB-)::mcjD | This work |
| BL21Ex1 | BL21D contain pSJ177 and pSJ221 | This work |
| BL21Ex2 | BL21D contain pSJ178 and pSJ221 | This work |
| BL21Ex3 | BL21D contain pSJ179 and pSJ221 | This work |
| BL21Ex4 | BL21D contain pSJ180 and pSJ221 | This work |
| BL21Ex5 | BL21D contain pSJ181 and pSJ221 | This work |
| BL21Ex6 | BL21D contain pSJ182 and pSJ221 | This work |
|  |  |  |
| Plasmids |  |  |
| pET28b | KanR, pBR322 ori |  |
| pDZcre | KanR, EmR, repA101, plem ori, expressing Cre enzyme and *E. coli*-Lactobacillus shuttle vector | Our lab |
| pDZcas | KanR, repA101, carrying arabinose induced λ-Red and constitutive expressed cas9 | Our lab |
| pT-empty | AmpR, pMB1 ori, promoter P2627 activates sgRNA  expression | Our lab |
| pSJex | AmpR, p15a ori, carrying promoter P2223 | Our lab |
| pTN52 | Fragment 1102FR inserts into pIN | This work |
| pSJ172 | Fragment *mcjA*-qPCR was inserted into pMD19 | This work |
| pSJ173 | Fragment *mcjB*-qPCR was inserted into pMD19 | This work |
| pSJ174 | Fragment *mcjC*-qPCR was inserted into pMD19 | This work |
| pSJ175 | Fragment *mcjD*-qPCR was inserted into pMD19 | This work |
| pSJ176 | pET28b contain fragment 47F36R | This work |
| pSJ177 | pET28b contain natural P_Q_-RBS-*mcjA* | This work |
| pSJ178 | pET28b contain natural P_Q_-RBS1-*mcjA* | This work |
| pSJ179 | pET28b contain natural P_Q_-RBS2-*mcjA* | This work |
| pSJ180 | pET28b contain natural P_Q_-RBS3-*mcjA* | This work |
| pSJ181 | pET28b contain natural P_Q_-RBS4-*mcjA* | This work |
| pSJ182 | pET28b contain natural P_Q_-RBS5-*mcjA* | This work |
| pThsds | The sgRNA of the *hsdS* gene is inserted into pT-empty | This work |
| pSJ221 | Contain P_2223_-*mcjBC* | This work |
| pSJ222 | Contain P_2223_-WT RBS-*mcjBCD* | This work |
| pSJ223 | Contain WT *mcjABCD* | This work |

**Table S2. Primers and PCR products used in this study**

| **Items** | | **Nucleotide sequence (5’-3’) description** | | **Purpose** | |
| --- | --- | --- | --- | --- | --- |
| **Primers** | |  | |  | |
| 1100F | | TATCGCGGCCGCGAGTTCACCACGGAACGCT | | amplification 1100FR | |
| 1100R | | GTCAGATCTATGCATTTGTCCCTTTACCG | |  |  |
| 1101F | | ACAGCTCGAGTCTGGCAACGGCACGAGC | | amplification 1101FR | |
| 1101R | | ATCGAGCTCATTAACGATGGTGAAGTAGA | |  |  |
| 1102F | | GAGTTCACCACGGAACGCT | | amplification 1102FR | |
| 1102R | | ATTAACGATGGTGAAGTAGA | |  |  |
| 1103F | | ATACAGGGAAAGTCCAGCT | | amplification 1103FR | |
| 1103R | | TGTGGTGCAACCTGGAGA | |  |  |
| 747F | | CTGAGATCTGATGGCTAAATATTCTG | | amplification 47F36R | |
| 749F | | ATACCCGGGCAGAATATTTAGCCATCA | | amplification 49F91R | |
| 972F | | GCAGGATCCGTCTGGCAAAATTCATTC | | amplification 72F91R | |
| 891R | | CATGTCGACTTATTCAGTAACAGAAGCCAG | |  |  |
|  | |  | |  | |
| 945F | | GCAGGATCCATTGAGTGTAAAGGCATAACTAC | | amplification 45F71R | |
| 971R | | TGAGTCGACTTAACCTTTATAATCAATG | |  |  |
| 836R | | GCTGTCGACTCAGCCATAGAAAGATATAG | |  | |
| 935F | | AGCTCTAGAACAGAAGGACGTGAGGT | | amplification 35F36R | |
| 936F | | CACTCTAGA**CAACTTTTATTCGACCCAAAGGGGGAAAAAT**ATGATTAAGCATTTTCAT | | amplification36F36R_ RBS1 | |
| 937F | | CACTCTAGA**CCCCCAATCAGAACCAAGTATAAGGAGGTTCAC**ATGATTAAGCATTTTCAT | | amplification37F36R_RBS2 | |
| 938F | CACTCTAGA**ACAATCCATCCCTTAAATACCAAGGAGGTCACCAC**ATGATTAAGCATTTTCAT | | amplification38F36R_ RBS3 | |  |
| 939F | CACTCTAGA**TCTACATACCACCCAAGTACTAAGGAGGTCCCAT**ATGATTAAGCATTTTCAT | | amplification39F36R_ RBS4 | |  |
| 940F | CACTCTAGA**CGCTCTAGGTAACAAAATAAGGAGGTCCATC**ATGATTAAGCATTTTCAT | | amplification40F36R_RBS5 | |  |
| HsdF | | ATCGGATCC**CTGGTGAACTTAACCATATA**GTTTTAGAGCTAGA | | amplification of HsdFR | |
| HsdR | | ATTACGCCAAGCTTGCATGCAGGC | |  | |
| **PCR products** | |  | |  | |
| Q | | TCGAGATCTCAATTCCGACGTCTAAGAAACCATTATTATCATGACATTAACCTATAAAAATAGGCGTATCACGAGGCCCTTTCGTCTTCACCTCGAGTCCCTATCAGTGATAGAGATTGACATCCCTATCAGTGATAGGGATACTGAGCACATCAGCAGGACGCACTGACCTCTAGACTG | | Synthesized promoter Q (Alper et al，2005) | |
| sgRNA | | GGATCCTTGACAGCTAGCTCAGTCCTAGGTATAATACTAGT**CTGGTGAACTTAACCATATA**GTTTTAGAGCTAGAAATAGCAAGTTAAAATAAGGCTAGTCCGTTATCAACTTGAAAAAGTGGCACCGAGTCGGTGCTTTTTTTCTGCAG | |  | |
| HsdFR | | 144bp BamHI/HindIII fragment containing hsdS’s sgRNA | | Cloning in pT-empty | |
| 1100FR | | 187bp NotI/BglII fragment containing hsdS’s left homologous arm | | Cloning in pIN | |
| 1101FR | | 198bp Xho/SacI fragment containing hsdS’s right homologous arm | | Cloning in pIN | |
| 1102FR | | 2195bp fragment containing leftarm-BBA_J23110-mcjD-rightarm | | For integration (Lambert et al，2007) | |
| 1103FR | | Positive integration 4727bp, negative 798bp | | Selecting positive and integration | |
| 72F91R | | 1810bp BamHI/SalI fragment containing mcjD gene | | Cloning in pIN | |
| 47F36R | | 443bp BglII/SalI fragment containing wt-mcjA gene | | Cloning pET28b | |
| 35F36R | | 261bp XbaI/SalIragment containing wt RBS and mcjA gene | | Cloning pDZ-Q | |
| 45F71R | | 2214bp BamHI/SalI fragment containing mcjBC genes | | Cloning pDZex | |
| 49F91R | | 4042bp SmaI/SalI fragment containing mcjBCD gene | | Cloning pDZex | |
| 45F91R | | 3973bp BamHI/SalI fragment containing RBS_wt-mcjBCD | | Cloning pDZex | |
| 36F36R | | 226bp XbaI/SalI fragment containing synthesized RBS1and mcjA gene | | Cloning pDZ-Q | |
| 37F36R | | 228bp XbaI/SalI fragment containing synthesized RBS2 and mcjA gene | | Cloning pDZ-Q | |
| 38F36R | | 230bp XbaI/SalI fragment containing synthesized RBS3 and mcjA gene | | Cloning pDZ-Q | |
| 39F36R | | 229bp XbaI/SalI fragment containing synthesized RBS4 and mcjA gene | | Cloning pDZ-Q | |
| 40F36R | | 226bp XbaI/SalI fragment containing synthesized RBS5and mcjA gene | | Cloning pDZ-Q | |

**Table S3. Qualitative and quantitative detection of biogenic MccJ25 by HPLC**

| Phase A: 100% water +0.1% TFA  Phase B: 90% acetone+ 0.1% TFA  Sampling volume: 10 μL  Current speed: 1mL/min  Wavelength: 254 and 280nm  Temperature: 25 ℃  Type of column: Agilent ZROBAX- 300SB, 5um, 4.6 × 150mm | | | | |
| --- | --- | --- | --- | --- |
| RT (min) | Phase A% | Phase B% |  |  |
| 4  6  8  10  20  22  27  27.01  35 | 90  70  60  50  50  0  0  90  90 | 10  30  60  50  50  100  100  10  10 |  |  |


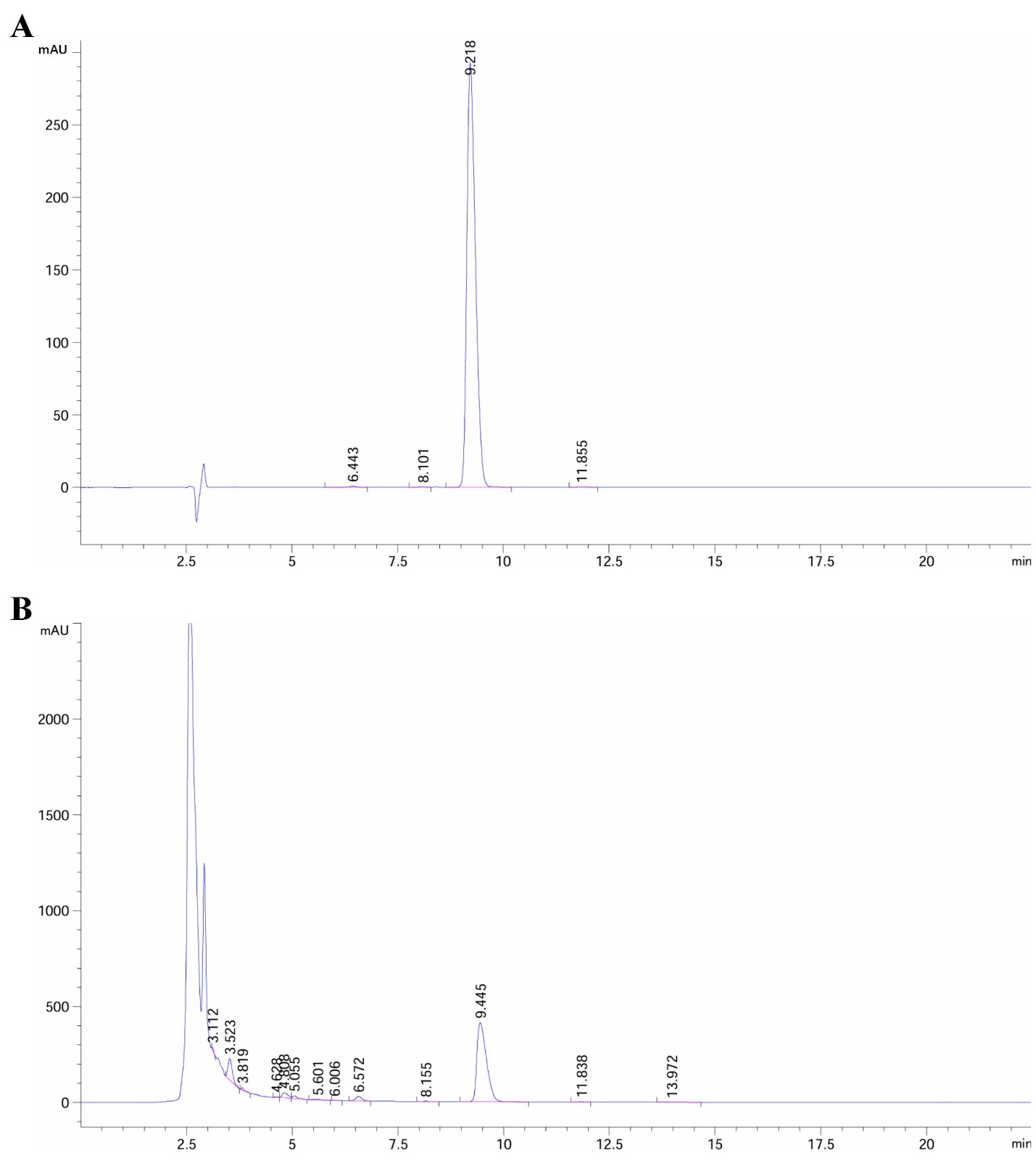


**Figure S1. MccJ25 in the fermentation broth of recombinant expression strains was detected by HPLC.** HPLC analysis of the MccJ25 (A) standard and (B) the fermentation broth of recombinant expression strains.


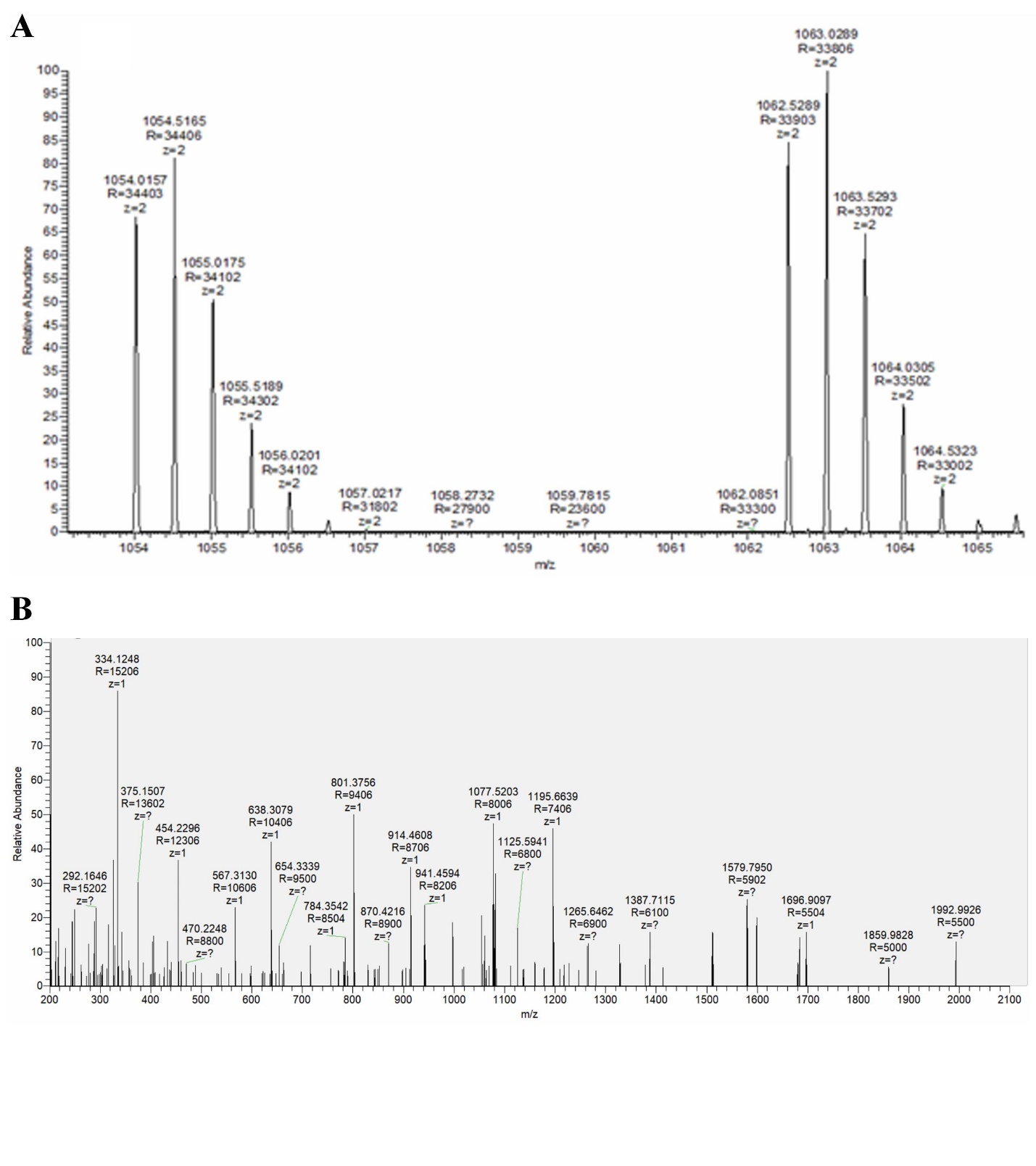


**Figure S2. LC-MS analysis of MccJ25 produced by recombinant expression strains.** (A) primary and (B) secondary mass spectra of MccJ25.


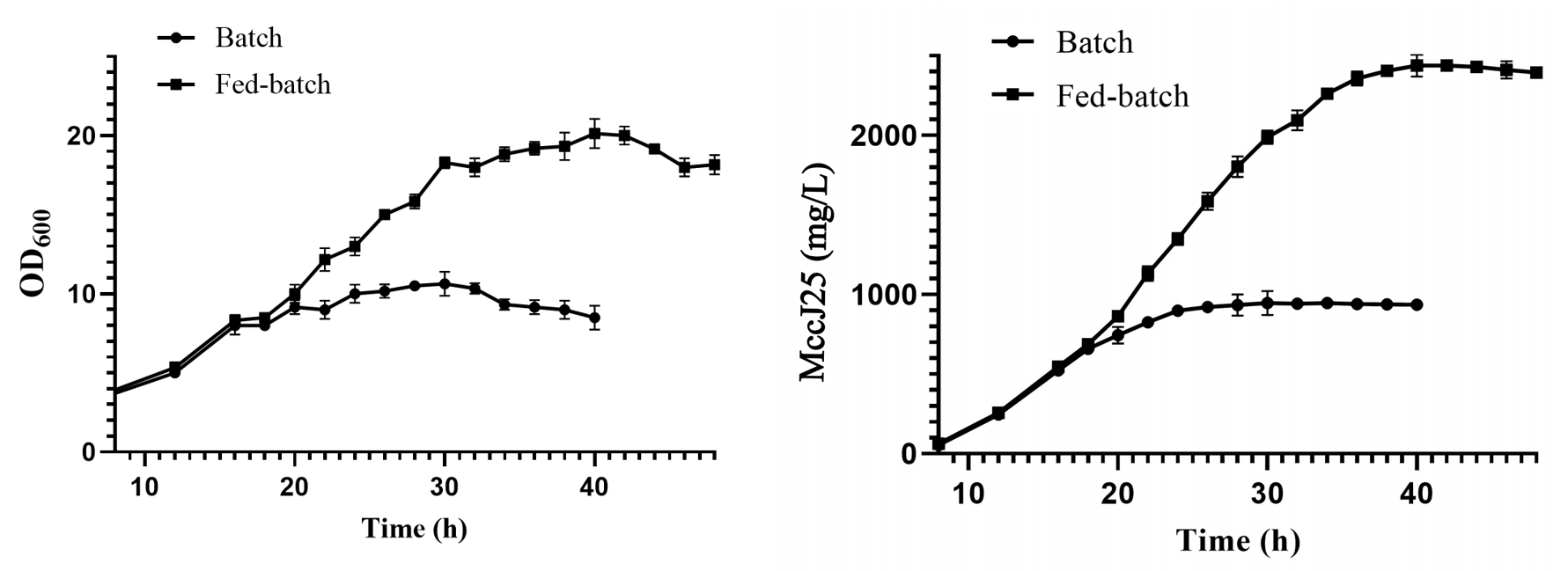


**Figure S3. Fed-batch cultivation enhances MccJ25 production in a 50 L bioreactor.** Time-course of MccJ25 production by genomically integrated strain BL21Ex4. The fermentation medium contained: 5 g/L sucrose, 20 g/L peptone, 25 g/L yeast extract, 2 g/L K₂HPO₄, 0.5 g/L MgSO₄·7H₂O, and 0.1 g/L MnSO₄·H₂O. Fermentation was performed in a 50 L bioreactor using 20 L working volume with 3% (v/v) inoculum, initial pH 6.5, and temperature 37°C; process parameters were maintained at pH 7.0 (automated regulation), 1.8 m³/h aeration, and 300 rpm agitation. Fed-batch supplementation employed a concentrated feed medium (glucose 26 g/L, peptone 205 g/L, yeast extract 263 g/L) controlled by pH-triggered logic (initiation at pH >7.0) with 20 mL/min flow rate (total feed volume 1.6 L), sampling every 30 s and 2 s response time. All experiments were independently repeated three times, data represents as mean ± SEM.
